# Hypoxic regulation of hypoxia inducible factor 1 alpha *via* antisense transcription

**DOI:** 10.1016/j.jbc.2023.105291

**Published:** 2023-09-23

**Authors:** Nicholas Downes, Henri Niskanen, Vanesa Tomas Bosch, Mari Taipale, Mehvash Godiwala, Mari-Anna Väänänen, Tiia A. Turunen, Einari Aavik, Nihay Laham-Karam, Seppo Ylä-Herttuala, Minna U. Kaikkonen

**Affiliations:** 1 A.I. Virtanen Institute, University of Eastern Finland, Kuopio, North-Savo, Finland; 2 School of Medicine, University of Eastern Finland, Kuopio, North-Savo, Finland; 3 Heart Center, Kuopio University Hospital, Kuopio, Finland

**Keywords:** hypoxia, lncRNA, HIF1a, transcriptional regulation, endothelial

## Abstract

Impaired oxygen homeostasis is a frequently encountered pathophysiological factor in multiple complex diseases, including cardiovascular disease and cancer. While the canonical hypoxia response pathway is well characterized, less is known about the role of noncoding RNAs in this process. Here, we investigated the nascent and steady-state noncoding transcriptional responses in endothelial cells and their potential roles in regulating the hypoxic response. Notably, we identify a novel antisense long noncoding RNA that convergently overlaps the majority of the hypoxia inducible factor 1 alpha (*HIF1A*) locus, which is expressed across several cell types and elevated in atherosclerotic lesions. The antisense (*HIF1A-AS*) is produced as a stable, unspliced, and polyadenylated nuclear retained transcript. *HIF1A-AS* is highly induced in hypoxia by both HIF1A and HIF2A and exhibits anticorrelation with the coding *HIF1A* transcript and protein expression. We further characterized this functional relationship by CRISPR-mediated bimodal perturbation of the *HIF1A-AS* promoter. We provide evidence that *HIF1A-AS* represses the expression of HIF1a in *cis* by repressing transcriptional elongation and deposition of H3K4me3, and that this mechanism is dependent on the act of antisense transcription itself. Overall, our results indicate a critical regulatory role of antisense mediated transcription in regulation of HIF1A expression and cellular response to hypoxia.

Hypoxia is typically defined as a relative reduction of oxygen supply that is insufficient to support normal physiological function. Since oxygen is an obligate requirement for aerobic metabolism in all multicellular organisms, a number of regulatory mechanisms have evolved to maintain oxygen homeostasis. Impaired oxygen homeostasis, or pathological hypoxia, is a frequently encountered pathophysiological factor in multiple complex diseases, including cardiovascular diseases, cancers, diabetes, and chronic lung diseases, which together account for 71% of all global deaths (1). The majority of the cellular hypoxic response is orchestrated by the hypoxia inducible factors (HIFs) that implement far reaching changes in the transcriptome to minimize oxygen consumption while supporting essential cellular functions and processes (2). The hypoxia response has been shown to alter the expression of a large subset of noncoding RNAs (3, 4), raising the possibility for their potential role in regulating aspects of the hypoxia-associated gene expression profile. Several well-characterized disease-associated long noncoding RNAs (lncRNAs), such as *MALAT1* (5), *H19* (6), and *WT1-AS* (7), have been shown to be differentially expressed in hypoxic conditions within various contexts, suggesting that the dysregulation of regulatory lncRNAs could also mediate a role in the etiology of diseases associated with hypoxia, such as chronic ischemic, inflammatory, and malignant neoplastic diseases (8, 9, 10, 11).

LncRNAs constitute a large proportion of the pervasively transcribed human genome. Given that lncRNAs are arbitrarily defined as RNA transcripts longer than 200 nucleotides that are not translated into proteins, this inclusion criteria results in a highly heterologous group of RNAs with diverse structural and presumably highly varied functional potential and characteristics. While over 50,000 lncRNA genes have been annotated within the human genome so far (12), only a minority have been functionally characterized and shown to be involved in a diverse array of processes, including cellular differentiation (13, 14), pluripotency (15, 16), and imprinting (14, 17, 18). Consequentially, the complete role, scope, and functional significance of lncRNAs within the cell regulatory framework remain largely enigmatic.

lncRNAs belonging to one of the largest subgroups, *cis*-regulatory lncRNAs, are expressed at relatively low steady-state levels, remain associated to the chromatin *via* RNAPII and exhibit relatively modest conservation compared to coding exons, yet slightly greater mean conservation than both introns and lncRNA exons (19), suggesting that they do not possess sequence-specific functionality. Unlike protein-coding mRNAs, the functionality of lncRNAs is not necessarily constrained to its encoded RNA sequence, instead various processes associated with its transcription and cotranscriptional processing can convey local regulatory activity on the expression of neighboring genes. For example, *cis*-acting lncRNA loci have been shown to function through transcriptional interference (14, 20, 21), transcriptional-dependent activation (22, 23), splicing (23), altered 3D genome structure (13) and altered epigenetic modifications (21, 24), among others. Due to the low steady-state expression of this class of lncRNAs, nascent RNA methodologies such as global run-on (GRO) and native elongating transcript sequencing are typically required to accurately map and quantitate sites of active transcription (25, 26). The limited number of well-characterized examples likely provide only a glimpse into what is perhaps a far more nuanced and pervasive form of regulatory interplay between *cis*-regulatory lncRNAs and neighboring coding-loci.

Due to the chromatin-conformational constraints on *cis*-regulation, the relative positioning and proximity of a *cis*-acting lncRNA loci to its neighboring genes is likely a key determinant of its activity. An estimated ∼40% of human protein-coding loci express overlapping natural antisense transcripts (NATs) with partial or total sequence complementarity (27, 28). While the efficient arrangement of overlapping transcriptional units is unsurprising in compact prokaryotic (29, 30) and eukaryotic (31, 32) genomes, its conservation within larger higher-order eukaryotic genomes suggests additional functionality. Despite the limited ability to use sequence conservation or mutagenesis to functionally characterize antisense lncRNAs, the prevalent and intrinsically linked arrangement of sense and antisense gene pairings represents an ideal basis to investigate putative *cis*-regulatory interactions with the potential to modulate gene expression. Several previously characterized NATs have been associated with transcriptional interference, where induced transcription of the NAT can repress the transcription of its cognate sense gene *in cis*. For example, transcription of the convergently transcribed antisense paternal *Airn* lncRNA through the *Igfr2* promoter silences the imprinted *Igf2r* cluster independently of the RNA transcript or general repressive chromatin marks (14). Alternatively, expression of the convergent antisense noncoding RNA in the INK4 locus at the *CDKN2B* locus in leukemia cell lines resulted in repression of *CDKN2B* mRNA expression through increased H3K9me2 and reduced H3K4me2 histone modifications at the *CDKN2B* promoter (24).

Here, we used genome-wide GRO sequencing (GRO-seq) and RNA-seq to identify noncoding and coding transcript antisense pairs that exhibit differential expression profiles under hypoxic conditions in primary human endothelial cells, and to investigate their regulatory potential. Unbiased *de novo* transcript identification revealed 2981 loci expressing antisense transcription, enabling a comprehensive insight into antisense transcriptional units under stimulus. Among these, only 25 loci displayed inverse regulation between the coding and noncoding transcripts. In particular, an overlapping lncRNA antisense to the HIF 1 alpha (*HIF1A*) locus showed robust and strong upregulation in response to hypoxia concomitant with downregulation of the *HIF1A* mRNA expression. This transcript was found expressed in multiple vascular cell types and upregulated in atherosclerotic femoral artery samples. Functional studies revealed that this novel transcript has a likely role in temporally regulating the expression of HIF1A in hypoxic conditions.

## Results

### The hypoxic noncoding transcriptome

**Figure 1:**
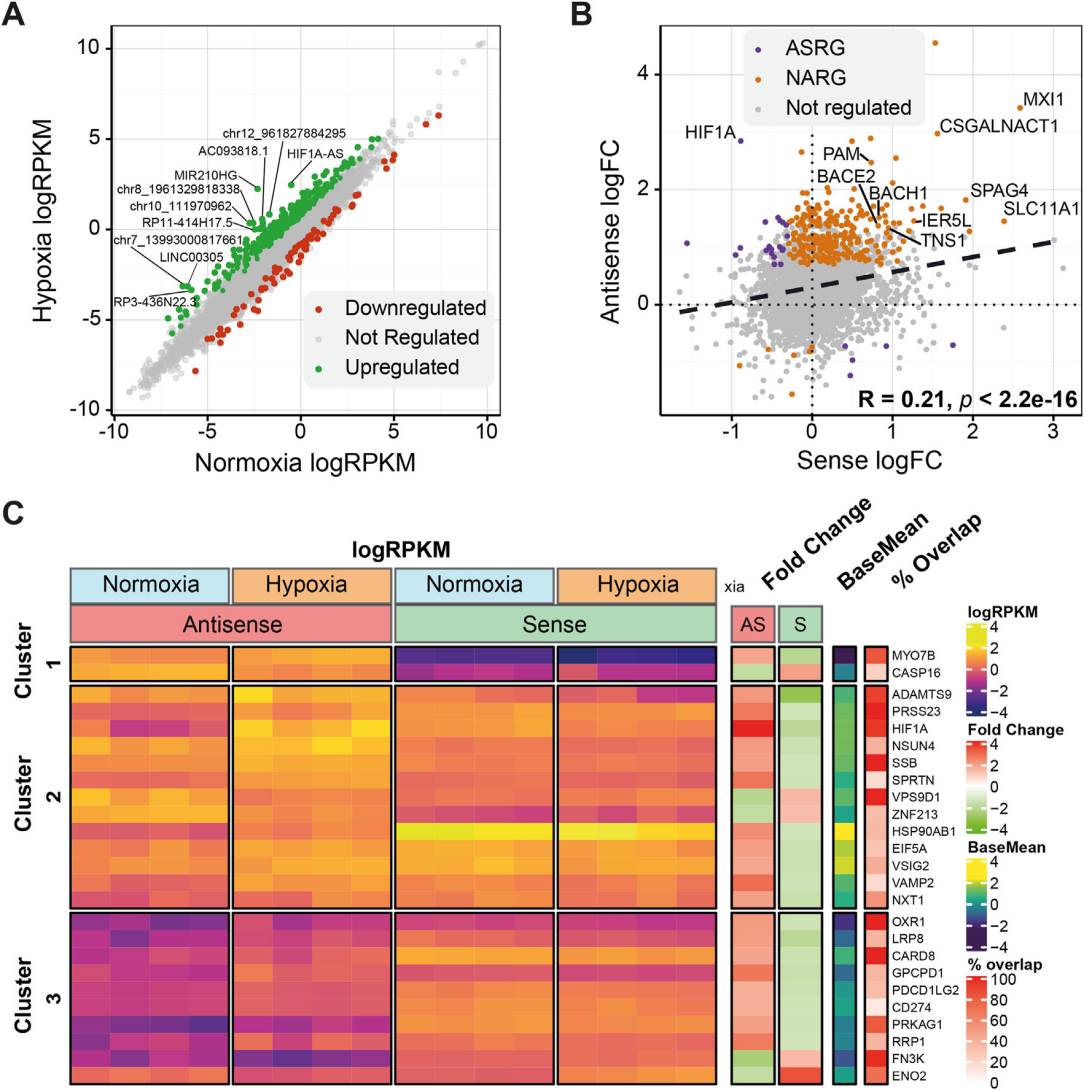
**Identification of hypoxia-regulated ncRNAs in HUVECs.***A*, expression of noncoding transcripts from HUVECs in hypoxia or normoxia given as log2 of reads per kilobase of transcript per million mapped reads (RPKM) values from GRO-seq, differentially expressed genes are colored with the top 10 differentially expressed genes annotated. *B*, correlation of sense and antisense gene pairs in hypoxia, loci are classified as antisense regulated genes (ASRG), nonantisense regulated genes (NARG) or not regulated. Log2FC = log2 fold change. *C*, heatmap of ASRG loci exhibiting inversely regulated sense and antisense expression, values given as k-means clustered log2 RPKM, log2 fold change, gene average expression values (logCPM baseMean) and sense/antisense overlap percentage. GRO, global run-on; HUVECs, human umbilical vein endothelial cells.

To identify novel pairs of noncoding NATs and coding genes we first identified lncRNAs transcriptionally regulated in hypoxia from GRO-seq performed in primary human umbilical vein endothelial cells (HUVECs) cultured under hypoxia for 8 h (1% O_2_) (3). GRO-seq captures nascent RNAs that are produced by elongation competent RNA polymerases, which is therefore useful for determining the rate and location of active transcription independently of transcript stability or posttranscriptional processing (33). We identified a total of 19,931 GENCODE V19 genes expressed in HUVECs, of which 2044 and 1928 genes were annotated as antisense and lncRNA, respectively. To further expand the list of potential hypoxia regulated transcripts, we performed *de novo* transcript identification to identify a further 5839 novel candidate transcripts expressed in HUVECs in either normoxic or hypoxic conditions. Of the 1281 genes differentially regulated in response to hypoxia, 457 were annotated as noncoding RNAs (ncRNAs) with 385 upregulated and 72 downregulated (Fig. 1*A*). Next, to identify those ncRNAs with the potential to regulate *in cis,* we focused on those orientated antisense to coding genes with a minimum of 10% sequence overlap. Altogether 2981 loci were seen to express antisense transcripts in HUVECs, with 272 being differentially expressed under hypoxic conditions. In general, antisense genes seemed to be loosely coregulated with their corresponding sense gene in hypoxia, as evidenced by the weak but significant positive correlation (*R* = 0.21, *p* < 2.2e-16) (Fig. 1*B*). Nevertheless, a subset of loci (n = 25) displayed inversely regulated sense and antisense expression patterns indicative of possible causative *cis* regulation (Fig. 1*C*).

### Identification of a convergent, hypoxia-inducible antisense RNA within the *HIF1A* locus

**Figure 2:**
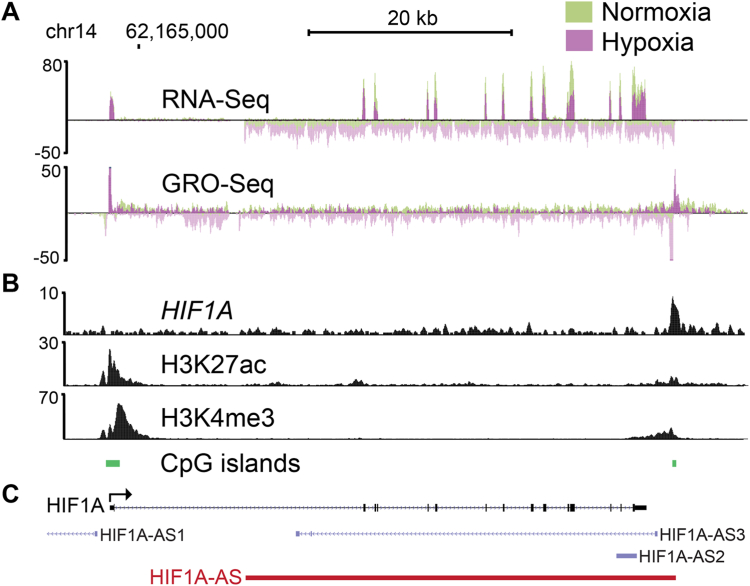
**Annotation and transcriptional and epigenetic signatures associated with the *HIF1A* and *HIF1A-AS* locus.***A*, UCSC genome browser (*http://genome.ucsc.edu**)* image of the locus (chr14:62,160,170–62,220,372 hg19). Normalized aligned reads for GRO- and RNA-seq for HUVECs treated with 7 h 1% O_2_ hypoxia or normoxia. *B*, public ChIP-seq for H3K27ac, H3K4me3, and HIF1A was analyzed to determine the putative promoter of *HIF1A-AS* and used to design flanking sgRNA sequences for CRISPR mediated ablation of *HIF1A-AS* expression. *C*, current annotations of *HIF1A-AS1* (NR_047116.1), *HIF1A-AS2* (NR_045406.1) and *HIF1A-AS3/HIFAL* (NR_144368.1). The *HIF1A-AS* characterized in this study is highlighted in *red* representing its annotation as an unspliced transcript that spans from its TSS at chr14:62,217,815 to its TTS at chr14:62,174,269. HIF, hypoxia inducible factor; ChIP, chromatin immunoprecipitation; GRO, global run-on; HUVECs, human umbilical vein endothelial cells; TSS, transcription start site; TTS, transcriptional termination site; sgRNA, single guide RNA.

In particular, the *HIF1A* locus expressed a highly induced antisense lncRNA (*HIF1A-AS*) in hypoxia which was accompanied by a 2-fold decrease in the expression of *HIF1A* nascent pre-mRNA (Fig. 2*A*). An equivalent level of repression was also seen at mature *HIF1A* mRNA levels (Fig. 2*A*). Further characterization of the *HIF1A-AS* locus revealed that it is initiated from a bidirectional transcription start site, feature common of most human genes (33, 34). *HIF1A-AS* was identified in both poly(A)+ selected and ribosomal-depleted RNAseq libraries, suggesting that the *HIF1A-AS* transcript is efficiently polyadenylated and relatively stable. The locus exhibited a clear enrichment for H3K4me3 and H3K27ac histone modifications approximately 2800 nt downstream of the *HIF1A* transcriptional termination site, and convergently overlaps the majority of the 53kb *HIF1A* locus (Fig. 2*A*). The *HIF1A-AS* promoter region (chr14: 62217554–62217813) was also seen to be well conserved in vertebrates as denoted by its region average phastCons100 score of 0.49. Screening for enriched transcription factor motifs within the *HIF1A-AS* promoter revealed a consensus hypoxia response element, which was confirmed to be bound by HIF1A in hypoxic conditions using public chromatin immunoprecipitation (ChIP)-seq data in HUVECs (35) (Fig. 2*B*). Latest NCBI RefSeq annotations reported three ncRNAs within this locus, corresponding to *HIF1A-AS1*, *-AS2* and *-AS3* (Fig. 2*C*). Notably, *HIF1A-AS3* showcased significant overlap with the *HIF1A-AS* characterized in this study. Yet, our analysis of the RNA-Seq data from HUVECs did not reveal major spliced variants of this transcript. A comprehensive examination of publicly available data from hypoxia-treated cancer cell lines, including HKC8, A549, H460, and RKO, affirmed our observations (Fig. S1). We thus report a long form of *HIF1A-AS* with widespread occurrence.

### HIFs modulate *HIF1A-AS* expression, leading to reduced HIF1A expression

**Figure 3:**
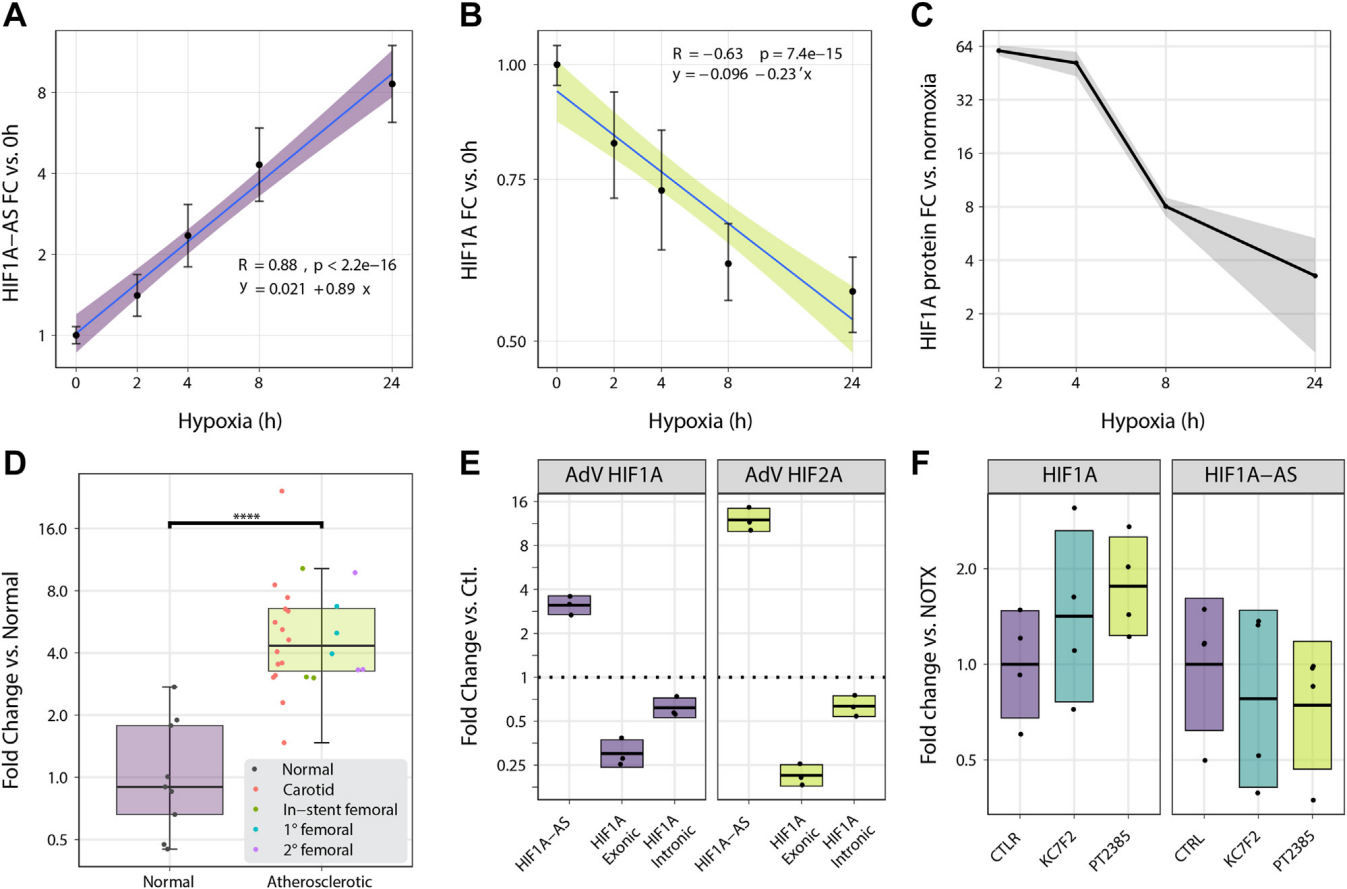
**Dynamics of *HIF1A* and *HIF1A-AS* expression during hypoxic stress.** Average relative expression of *HIF1A-AS* (*A*) and *HIF1A* (*B*) during 0 to 24 h 1% O_2_ hypoxia in HUVECs determined as by qPCR. Pearson correlation coefficient *R* = 0.88 and *R* −0.63, *p* = < 2.2 × 10^−16^ and 7.4 × 10^−15^, respectively. Error bars represent 95% confidence intervals (n = 3). *C*, the quantification of HIF1A protein in HUVECs was assessed using Western blotting after exposing the cells to various durations of hypoxia, compared to normoxia. The *gray* area in the data visualization represents the standard deviation (n = 2). *D*, qPCR quantification of median *HIF1A-AS* expression in human atherosclerotic femoral artery samples (n = 24) compared with normal internal thoracic artery (n = 9), *p* < 1 × 10^−5^, two sample *t* test. Error bars represent 1.5 times interquartile range. *E*, average RNA-seq quantification of intronic and exonic *HIF1A* transcripts, and *HIF1A-AS* transcripts after adenoviral (AdV) overexpression of stabilized forms of HIF1a and HIF2a in HUVECs (n = 3). Crossbar range represents standard deviation. *F*, quantification of *HIF1A* and *HIF1A-AS* RNA in HUVECs following treatment with the isoform specific HIF1A (KC7F2) and HIF2A (PT2385) inhibitors relative to no treatment (NOTX) (n = 4). *Line* represents mean, and standard deviation is shown by the *crossbar* range. HIF1A, hypoxia inducible factor 1 alpha ; HUVECs, human umbilical vein endothelial cells; qPCR, quantitative PCR.

To ascertain how the expression dynamics of *HIF1A* and *HIF1A-AS* changes in relation to each other following the onset of hypoxia, we performed a time-course study to quantitate and correlate their relative expression changes over time by quantitative reverse transcription PCR. The results demonstrated 2-fold induction of *HIF1A-AS* as early as 2 h after the onset of hypoxia that continued to accumulate reaching more than 10-fold after 24 h exposure (*r* = 0.88), paralleled by a concurrent reduction in HIF1a transcript levels over time (*r* −0.63; Fig. 3, *A* and *B*). At the protein level, HIF1A induction was observed at the 2 h and 4 h time points, consistent with rapid protein stabilization (Figs. 3*C* and S2). However, these levels swiftly declined between 8 to 24 h, mirroring the changes seen in mRNA levels.

Concurrent hypoxic expression patterns of *HIF1A-AS* and *HIF1A* was also observed in human aortic endothelial cells (HAECs), CD14+ monocytes, and aortic smooth muscle cells (Fig. S3). Although most prominent in hypoxia, there was also evident anticorrelation between the nascent expression of *HIF1A* and *HIF1A-AS* under basal conditions in 12 different cell types (Fig. S4). Atherosclerotic plaques are comprised of a variety of vascular cell types and frequently exhibit hypoxic regions, especially in the atheromatous core (36). Quantitation of *HIF1A-AS* expression in atherosclerotic lesions obtained from patients undergoing femoral endarterectomy surgery for peripheral artery disease revealed an average 9-fold (*p* < 0.0001) higher expression of *HIF1A-AS* when compared with normal internal thoracic arterial tissue (Fig. 3*D*). *HIF1A-AS* was upregulated in all atherectomy subtypes with no difference observed between restenotic or primary atherosclerotic lesions. These results suggest that *HIF1A-AS* is expressed from multiple vascular cell types and the induction of the *HIF1A-AS* but also a physiological response to hypoxia stimulus *in vivo*.

To further clarify the role of HIFs in the hypoxic induction of *HIF1a-AS* transcription, we transduced HUVECs with adenovirus constructs overexpressing stabilized forms of HIF1A and HIF2A in normoxic conditions (37). Both HIF1A and HIF2A increased the expression of *HIF1A-AS* 10-fold and 45-fold respectively, relative to cells transduced with a control vector–indicative that the hypoxic induction of *HIF1A-AS* transcription is likely mediated by HIFs. Similarly, expression of stabilized HIF1A and HIF2A with subsequent *HIF1A-AS* induction resulted in repression of endogenous *HIF1A* pre-mRNA and mRNA expression as ascertained from intronic and exonic reads (Fig. 3*E*). Conversely, the use of specific HIF1A (KC7F2) and HIF2A (PT2385) inhibitors showed a decrease in *HIF1A-AS* expression and an increase in *HIF1A* mRNA, providing further corroborative evidence (Fig. 3*F*). The ability for HIF1A to induce transcription of the *HIF1A-AS* under hypoxic conditions, which in turn appears to repress expression of *HIF1a* mRNA suggested that *HIF1A-AS* may function as part of a negative-feedback circuit.

### *HIF1A-AS* regulates the expression of *HIF1A* through *cis*-acting *transcriptional* control

**Figure 4:**
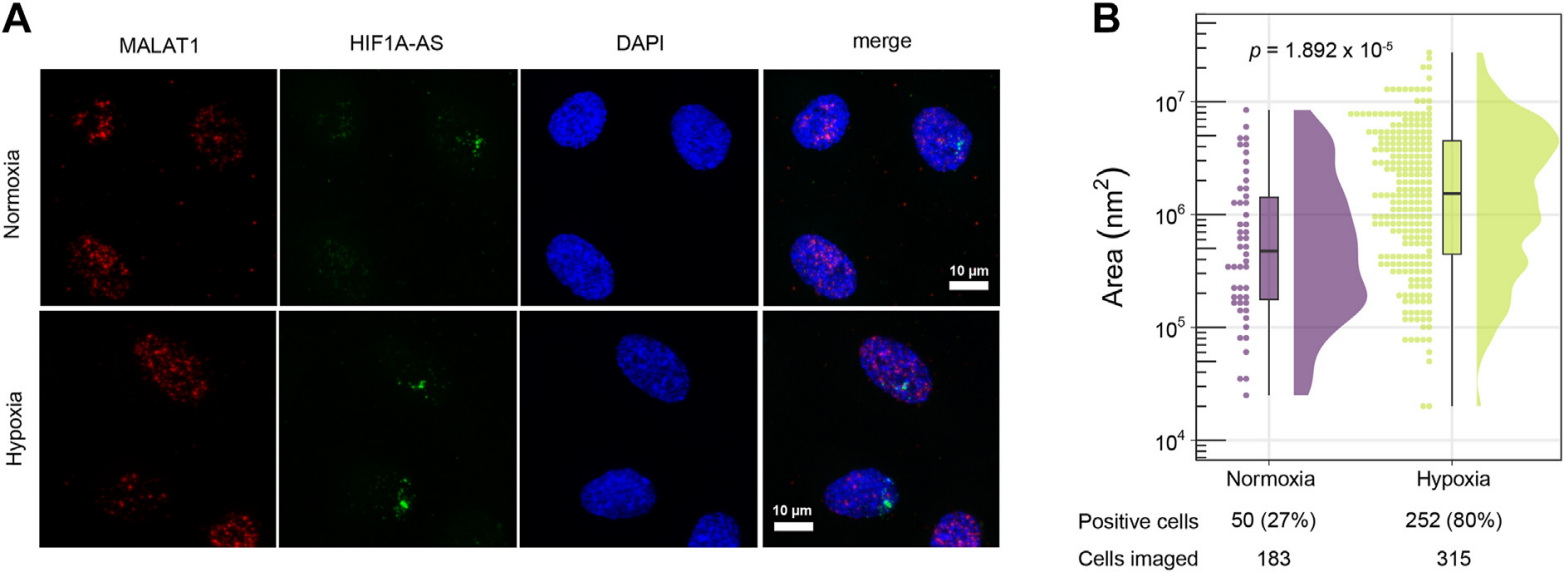
**Nuclear localization of *HIF1A-AS* in HUVECs.***A*, smRNA FISH showing nuclear localization of *HIF1A-AS* (*green*) in HUVECs. MALAT1 (*red*) was used as a positive control for nuclear RNA staining. *B*, quantification of nuclear area with RNA FISH signal in normoxic and hypoxic cells. Points represent individual nuclei, box plot represent median, 25th and 75th percentiles. *p*-value (1.892 × 10^−5^) is from Wilcoxon rank sum test. smRNA, small RNAs; HIF1A, hypoxia inducible factor 1 alpha; HUVECs, human umbilical vein endothelial cell.

The extensive anticorrelation between *HIF1A-AS* and *HIF1A* in hypoxia prompted us to further investigate the mechanism underlying this regulation. To see if the *HIF1A-AS* transcript was localized to a nucleus in line with a potential role as a transcriptional regulator, we first characterized the cellular distribution and localization of the *HIF1a-AS* transcript using single molecule RNA FISH. Imaging revealed that the *HIF1a-AS* transcript predominantly localized to 1 to 2 distinct foci within the nucleus, in line with a *cis*-acting lncRNA (Fig. 4*A*). Quantitation of the small RNA-FISH signal also confirmed an average 5-fold increase in *HIF1a-AS* levels in hypoxic conditions (Fig. 4*B*).

**Figure 5:**
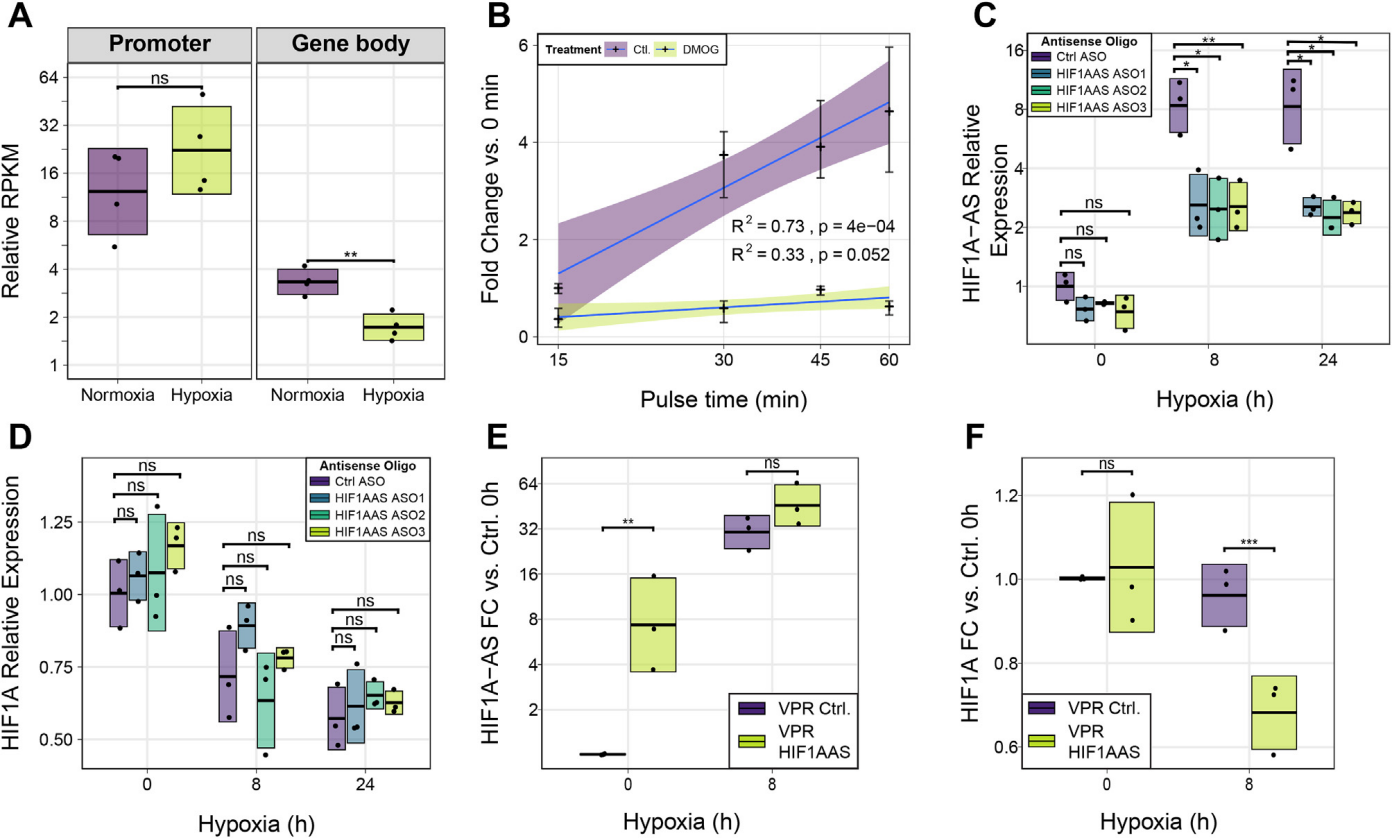
P**erturbation of *HIF1A-AS* expression points to a *cis*-regulatory mechanism.***A*, relative quantification of GRO-Seq signal from four HUVEC replicates between normoxia and hypoxia from the *HIF1A* promoter-proximal region (0–300 bp from TSS) (*p* = 0.227 - two sample *t* test) and from the *HIF1A* gene body (*p* = 0.00253 - two sample *t* test). *Line* represents mean and standard deviation is shown by the *crossbar* range. *B*, relative expression of *HIF1A* pre-mRNA labeled with 5-ethynyl uridine for 15, 30, 45, or 60 min in untreated and dimethyloxallyl glycine (DMOG) treated HUVECs, error line represents 95% confidence intervals. Slope *P =* 4 × 10^−4^. *C*, knockdown of *HIF1A-AS via* RNase-H mediated ASOs in normoxia, 8 h and 24 h hypoxia in HUVECs (n = 3). ns = not significant, ∗ = adj.*p* < 0.05, ∗∗ = adj.*p* <0.01 (*t* test with Bonferroni correction). *D*, ASO knockdown of HIF1a-AS does not significantly alter HIF1a mRNA expression in HUVECs. ns = not significant, (*t* test with Bonferroni correction). *E*, induced expression of *HIF1A-AS* using VPR with gRNA targeting the putative *HIF1A-AS* promoter in normoxic and hypoxic HEK 293T cells (*p* = 0.00527, *t* test, n = 3). *F*, induced expression of *HIF1A-AS via* dCas9-VPR reduced *HIF1A* mRNA expression in hypoxic 293T cells (*p* = 0.000876, *t* test, n = 3). ASO, antisense oligonucleotides; gRNA, guide RNA; HIF1A, hypoxia inducible factor 1 alpha; GRO, global run-on; HUVECs, human umbilical vein endothelial cells; TSS, transcription start site.

Since transcriptional regulation can be exerted through reduced RNAPII initiation events or decreased RNAPII pause-release, GRO-seq reads were binned to proximal-promoter and gene-body regions of the *HIF1A* locus in normoxic and hypoxic conditions (Fig. 5*A*). In hypoxia, levels of nascent RNA are reduced in the *HIF1A* gene-body while increased in the proximal-promoter, suggesting that induced RNAPII proximal-pausing and decreased transcriptional elongation may contribute to reduced *HIF1A* expression. These results were further confirmed using metabolic pulse-labeling of nascently transcribed RNA with 5-ethynyl uridine over several time points (approach-to-equilibrium). Treatment of HUVECs with dimethyloxallylglycine, a competitive inhibitor of HIF-hydroxylated prolyl hydroxylase, resulted in a significantly reduced transcriptional rate of *HIF1A* pre-mRNA than the untreated cells (Slope 0.34 *versus* 1.91, *p* =0.0018; Fig. 5*B*), and a corresponding increase in *HIF1A-AS* transcription (Fig. S5). Altogether, these results suggest that the induction of *HIF1A-AS* correlates with reduced transcriptional elongation of *HIF1A* and could represent a mechanism to buffer *HIF1A* expression.

To clarify a causal *cis*-regulatory link between *HIF1A-AS* and *HIF1A* transcription, we next sought to perturb the expression of *HIF1A-AS* in order to elucidate the mode of regulation it may have on *HIF1A*. lncRNAs typically act in-*cis* through either the process of transcription of the lncRNA (23, 38) or through the RNA transcript (17, 23). To initially explore these two mechanisms, three different antisense oligonucleotides (ASO) were transfected in HUVECs to knockdown the steady-state levels of *HIF1A-AS via* RNase-H, resulting in approximately 70% knockdown relative to scrambled controls (Figs. 5*C* and S6). Despite the reduction in the levels of *HIF1a-AS*, the hypoxia-associated reduction in *HIF1A* expression remained largely unchanged (Fig. 5*D*). Subsequently, we next sought to bimodally perturb the transcription of *HIF1A-AS* using CRISPR approaches and to ascertain any effects it may have on *HIF1A* expression. In order to drive the ectopic expression of *HIF1A-AS*, a catalytically dead Cas9 (dCas9) fused to a tripartite of transcriptional activators (VPR ie, VP64, p65 and Rta) (39) specifically targeting the *HIF1A-AS* promoter was used. VPR together with a *HIF1A-AS* targeting guide RNA (gRNA) in 293T cells resulted in a 5-fold induction of *HIF1A-AS* in normoxia, and a 1.5-fold induction in hypoxia (Fig. 5*E*). The lower VPR-mediated induction of *HIF1A-AS* in hypoxia could be due to the already strong hypoxia-mediated induction of *HIF1A-AS* in these cells or steric competition between HIF1/2A binding and dCas9-VPR (Fig. 2*C*). Importantly, the VPR-mediated 1.5-fold induction of *HIF1A-AS* in hypoxia was concurrent with a 1.5-fold repression of *HIF1A* mRNA when compared with no gRNA controls (*p*-value ≤ 0.05, Welch's *t* test; Fig. 5*F*). Unexpectedly, we observed no discernible effect under normoxic conditions. Collectively, our data indicate that *HIF1A-AS* may modulate the expression of *HIF1A* in *cis via* a transcription-mediated mechanism particularly during hypoxia.

### Ablation of HIF1a-AS expression is associated with changes in H3K4me3 at the *HIF1A* promoter

**Figure 6:**
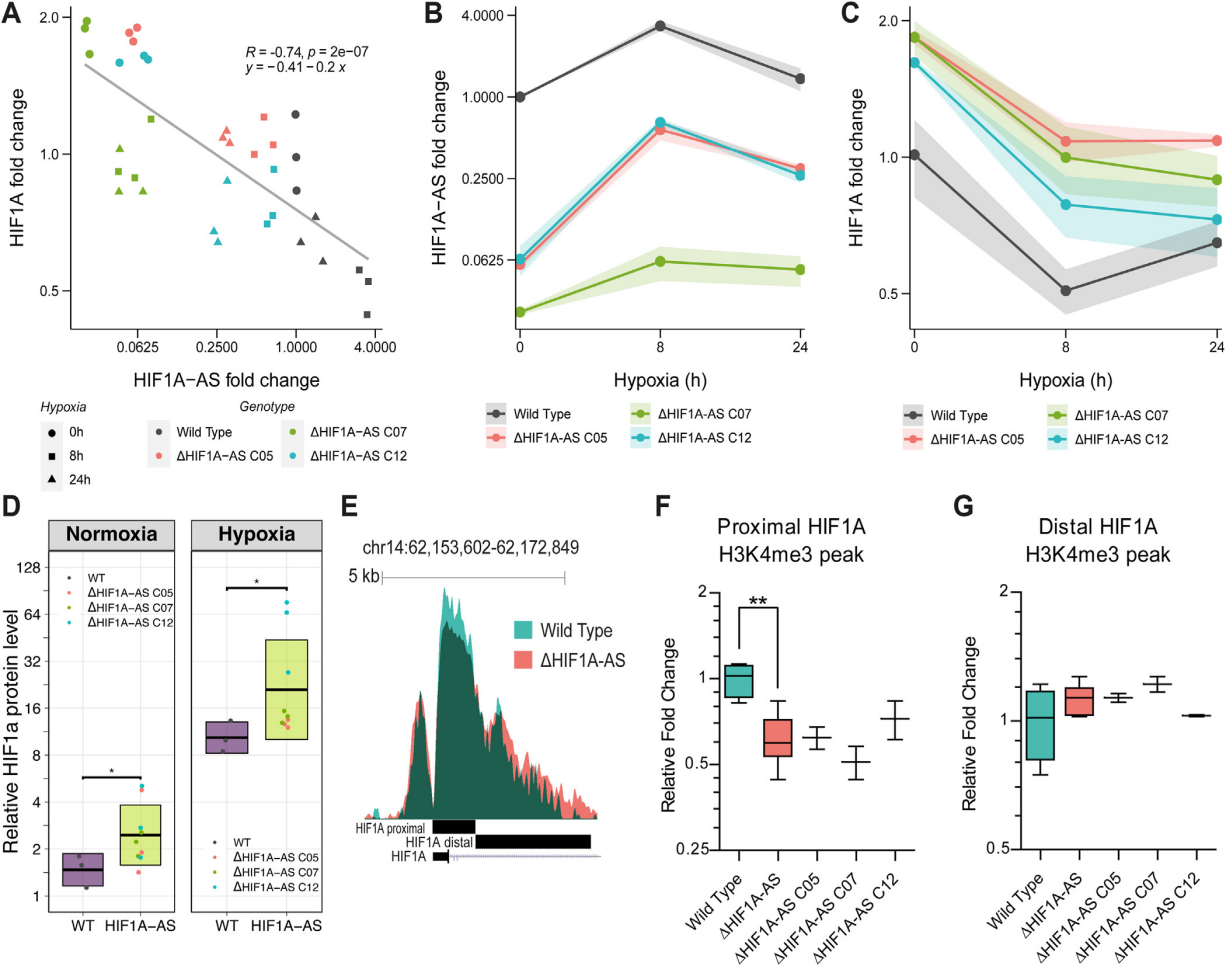
**Impacts of *HIF1A-AS* promoter ablation on *HIF1A* expression and epigenetic regulation.***A*, ablation of the *HIF1A-AS* promoter using Cas9 CRISPR editing in EAhy cells (*ΔHIF1A-AS*) depletes HIF1a-AS expression which exhibits strong anticorrelation with HIF1a expression in both normoxic and hypoxic conditions (*R =* −0.74) (n = 3). Three clones, named C05, C07, and C12 were produced and evaluated in comparison to WT cells. *B*, *ΔHIF1A-AS* cells express HIF1A-AS at consistently lower levels in normoxic and hypoxic conditions relative to WT cells. *C*, *ΔHIF1A-AS* EAhy cells exhibit consistently higher relative expression of HIF1a in normoxia and hypoxia compared with WT controls, points given as an average from three replicates with standard deviation represented by the shaded ribbon. *D*, Western blot quantification of HIF1a protein concentrations from WT and *ΔHIF1A-AS* EAhy cells in normoxia (*p* = 0.0391, *t* test) and hypoxia (*p* = 0.0292, *t* test), sine represents mean and standard deviation is shown by the crossbar range (n = 3). *E*, UCSC track showing normalized H3K4me3 ChIP-seq reads from the *HIF1A* promoter region, where *ΔHIF1A-AS* cells exhibit lower H3K4me3 peak height at the *HIF1A* proximal promoter region immediately downstream of the TSS (*F*) but exhibit a broader H3K4me3 peak profile with higher levels of occupancy in the distal region (*G*). ChIP, chromatin immunoprecipitation; HIF1A, hypoxia inducible factor 1 alpha; TSS, transcription start site.

To further investigate the functional mechanism by which *HIF1A-AS* could regulate *HIF1A* expression, we ablated *HIF1A-AS* expression by removing its core promoter (Fig. S6) with high fidelity Cas9 to create clonal EA.hy926 cell lines (*ΔHIF1A-AS,* n = 3). Removal of the 480 bp promoter region resulted in an average 30- and 15-fold reduction in *HIF1A-AS* expression in normoxia and hypoxia, respectively (Fig. 6, *A* and *B*). Consistent with data presented above, ablation of *HIF1A-AS* expression resulted in an average 2-fold increase in HIF1A mRNA and protein expression in hypoxia when compared with WT control cells (Figs. 6, *A* and *C* and S7). This was accompanied with stronger induction of hypoxia-related pathways in ΔHIF1A-AS cells (Fig. S8) confirming the functional impact on hypoxic HIF-1 response. Interestingly, the ΔHIF1A-AS cells maintained a constantly higher expression of *HIF1A* that was largely independent of hypoxia. In line with this, the paired expression of *HIF1A* and *HIF1A-AS* maintained a robust anticorrelation (*r* = −0.74) in all conditions (Fig. 6*A*).

While convergent transcription has been associated with transcriptional interference and reduced transcriptional output, antisense and convergent transcription has also been seen to alter promoter chromatin states (40). Higher levels of the promoter associated H3K4me3 histone demarcation is associated with genes exhibiting high convergent antisense expression (40). To this end, we sought to ascertain if the deposition of the H3K4me3 mark was altered at the *HIF1A* promoter as a result of ablated *HIF1A-AS* expression in hypoxia. Our results demonstrated significant repositioning of the H3K4me3, associated with reduced proximal H3K4me3 levels around the transcription start site of *HIF1A* but increased H3K4me3 occupancy and peak width at distal sites upstream and downstream of the *HIF1A* promoter (Fig. 6, *E*–*G*). Additionally, we examined the alterations in H3K27ac, indicative of transcriptional activity, and H3K36me3, representative of elongation. Both of these marks decreased noticeably around the *HIF1A-AS* promoter upon its deletion. However, this resulted in only subtle regional alterations in H3K36me3 within the *HIF1A* gene body and no discernible change in H3K27ac at its promoter (Fig. S9). Altogether, these data suggest that *HIF1A-AS* transcription could be involved in repression *HIF1A* transcription in a process that potentially involves H3K4me3 chromatin remodeling.

## Discussion

Up to 40% of human protein-coding loci exhibit the presence of overlapping NATs (27, 28). Analyses of genome-wide expression patterns within sense-antisense pairs consistently reveal a prevalent positive correlation whereas instances of inverse correlation appear less frequent (27, 28, 41). Our study aligns with this trend, with only ∼1% of lncRNAs demonstrating significant inverse regulation. Among the identified hypoxia-responsive NATs showing inverse regulation, *ADAMTS9-AS1/2* (42, 43, 44), *VPS9D1-AS1* (45, 46), *CARD8-AS1* (47), and *PRKAG1* antisense RNA 1 (48) have been previously described. However, all these studies have primarily focused on the transcript-mediated roles, especially through miRNA sponging activity associated with tumor progression. In this work, we identify and characterize a novel *HIF1A-AS* transcript that exhibits an inverse correlation with *HIF1A* coding gene expression. This correlation arises from the transcriptional repression of both *HIF1A* pre-mRNA and mRNA, which subsequently translates into decreased protein levels.

Using unbiased nascent and steady-state sequencing approaches combined with *de novo* transcript identification, we were able to show that a convergent, unspliced, *HIF1A-AS* transcript overlaps the majority of the *HIF1A* locus in multiple primary cell types. Due to the absence of significant splicing events identified within *HIF1A-AS*, we proceeded to investigate the role of the full-length *HIF1A-AS* transcript. We observed that *HIF1A-AS* represses *HIF1A* expression through *cis* transcriptional mechanisms which is supported by the following: (i) induction of *HIF1A-AS* equally represses *HIF1A* pre-mRNA expression to the same extent as the mature mRNA; (ii) the nuclear retention of the *HIF1A-AS* transcript (in contrast to mRNA destabilizing effects widely taking place in cytoplasm); (iii) derepression of *HIF1A* expression following ablation of *HIF1A-AS* transcription, which was not seen with posttranscriptional mediated ASO knockdown of the *HIF1A-AS* RNA.

While the mechanisms behind *cis*-NAT regulation are currently poorly understood, several hypotheses have been proposed. These include transcript-dependent mechanisms such as double stranded RNA formation and RNAi (49), lncRNA-directed epigenetic modifications, such as DNA methylation (21), changes in chromatin state and histone modifications (50, 51) and regulation of alternative splicing (52). Independent of AS-transcript itself, AS-transcription may lead to transcriptional interference (53), for example, when convergent transcription of the sense and AS results in polymerase collision and premature termination (54, 55)–an effect that is also observed at actively transcribed intragenic enhancers (20). Here, we observed that ablation of *HIF1A-AS* resulted in the redistribution of histone H3 methylation patterns at the *HIF1A* promoter, suggesting that convergent antisense transcription could be linked to some extent changes in the H3K4me3 enrichment at the sense promoter. It has been well established that both H3K4me3 peak height and peak breadth correlate positively with gene expression and H3K4me3 increases over genes during their transcription induction (56). Still, it is unclear if the deposition and maintenance of H3K4me3 at the sense promoter is a cause or consequences of reduced sense transcription under conditions of high antisense transcription. However, it is possible that HIF1a-AS dependent reduction in elongating RNAPII at the *HIF1A* promoter may inhibit elongation dependent recruitment of H3K4me3-depositing complexes such as chromodomain helicase DNA binding protein 1, thus narrowing the H3K4me3 peak (57). In addition, transcription of the AS-strand has been shown to influence H3K36me3 deposition and histone deacetylase recruitment at mRNA promoters, reducing the expression of the sense strand (58, 59). However, only small local changes in H3K36me3 were seen which could be attributed to a maintained equilibrium in the overall transcriptional activity of sense-antisense transcription at the locus. On the other hand, the absence of change in H3K27ac at *HIF1A* promoter supports the notion that the effect of *HIF1A-AS* occurs post-initiation. Further investigation is thus required to understand the role of H3K4me3 changes at the gene promoter and the exact molecular mechanisms mediating the repression of *HIF1A* transcriptional elongation.

The *HIF1A* locus has previously been observed to express a shorter, convergent antisense transcript (termed HIF1A-AS2, NR_045406.1) that partially overlaps the 3′ UTR using differential display (60). The spliced 2051 nt transcript was shown to be highly associated with malignant cancers, such as nonpapillary renal cell carcinoma, breast cancer, and glioblastoma (60, 61, 62, 63), as well as ischemic conditions, such as myocardial infarction (64, 65). *In silico* predictions suggested that the *HIF1A-AS2* hybridizes to and exposes an AU-element in the *HIF1A* 3′UTR, leading to subsequent destabilization (66). However, this evidence has not been supported by dsRNA ligation assays *in vitro* (67), and in glioblastoma cells the *HIF1A* mRNA levels remained stable during hypoxia despite significantly upregulated *HIF1A-AS2* (62). In addition, *HIF1A-AS2* has been proposed to mediate other *trans* effects on genes involved in hypoxic adaptation, maintaining pluripotency, and possibly contributing to tumorigenesis of glioblastoma (62). *HIF1A-AS2* has also been proposed to function as a sponge for miR-153-3p in HUVECs, and thus hypoxia-induced *HIF1A-AS2* upregulation could repress the degradation of *HIF1A* transcript (68). The extensive length of *HIF1A-AS* identified in this study would make exclusive hybridization to the *HIF1A* 3′UTR for posttranscriptional regulation in the cytoplasm energetically and spatially unfavorable. Still, our results do not exclude the possibility for the existence of a spliced transcript that could mediate differential *trans* effects in transformed and other cell types not investigated here. Supporting this, the latest GENCODE annotations emerged during the preparation of this manuscript now recognize the long lncRNA transcript described here as *HIF1A-AS3* and propose existence of additional spliced forms. To this end, a recent report by Zheng *et al*, demonstrated that *HIF1A-AS3* locus does produce 659 nucleotides long spliced *HIF1A* anti-sense lncRNA, *HIFAL*, in breast cancer cells which induces HIF1A *trans*-activation through recruitment of prolyl hydroxylase 3 (63). On the other hand, Ma *et al.* predicted a distinct six-exon spliced lncRNA based on human expressed sequence tag data. They demonstrated that transcriptional silencing of the locus using oligo phosphodiester nucleotides led to the repression of *HIF1A* expression through a *cis*-mediated mechanism in HUVECs (69). This finding not only lends further support to our own results but also suggests that prior investigations may have overlooked the potential role of the full-length *HIF1A-AS* transcript in mediating these effects. In fact, our analysis of *HIF1A-AS* expression across several other cell types revealed that the major transcript could indeed exist as a full-length unspliced isoform. All this evidence calls for further investigation to understand the expression profiles of the full-length and spliced forms of *HIF1A-AS* and their associated functions across different cell types and conditions.

Interestingly, the hypoxic expression of *HIF1A-AS* was largely shown to be regulated by HIF1A and HIF2A, suggesting *HIF1A-AS* acts as part of a negative-feedback system. Since HIF1A is associated with the acute hypoxia-response, and HIF2A with the more chronic adaptive responses (37, 70, 71), it is possible that *HIF1A-AS* is induced by HIF2A to enforce a limited temporal expression profile of HIF1A after prolonged periods of hypoxia. As an indication of the physiological relevance of our findings, the expression of *HIF1A-AS* was also highly upregulated in human atherosclerotic vascular samples often characterized by their hypoxic environment (36) when compared with normal tissue. Hypoxia and HIF1A have been recognized to be important contributors to progression of atherosclerosis, as they can participate to endothelial dysfunction and stimulate inflammatory and angiogenic responses within the lesion (72, 73, 74).

In conclusion, we demonstrate that *HIF1A* locus exhibits extensive AS-transcription upon hypoxia in vascular cells, directly regulating *HIF1A* at the transcriptional level. These discoveries open up new mechanisms in explaining the regulation of *HIF1A* in response to hypoxia.

## Experimental procedures

### Cell culture

HUVECs were isolated from cords obtained from the Kuopio University Hospital with the approval from the Research Ethics Committee of the Northern Savo Hospital District HUVECs from three individual donors were pooled and cultured in endothelial growth medium (EGM BulletKit; Lonza) in plates coated with 10 g/ml fibronectin (Sigma-Aldrich) and 0.05% gelatin in PBS. HUVECs were cultured up to passage 7. HAECs (CC-2535, Lonza) were grown in similar conditions as HUVECs. Human aortic smooth muscle cells (HASMCs, #C-007-5C, Gibco) were grown in Medium 231 (#M-231–500, Gibco) supplemented with Smooth Muscle Growth Supplement (S-007–25, Gibco). CD14+ macrophages were differentiated from CD14+ monocytes (#2W-400 C, Lonza), that were cultured in RPMI 1640 supplemented with 10% fetal bovine serum, antibiotics (100 U/ml penicillin/100 μg/ml streptomycin (#P0781, Sigma-Aldrich), 2 mM glutamine, 1% Na-pyruvate, 1% nonessential amino acids and recombinant human M-CSF (50 ng/ml, RP-8643, Invitrogen) until cells had adhered (minimum 7 days). hTERT-immortalized human aortic endothelial cells (TeloHAECs) were cultured using vascular cell basal medium (PCS-100–030, ATCC) supplemented with Vascular Endothelial Cell Growth Kit-VEGF (PCS-100–041, ATCC) and penicillin/streptomycin as above. HEK293T and EA.hy926 cells were cultured in Dulbecco's modified Eagle's medium (#D6429, Sigma-Aldrich) supplemented with 10% fetal bovine serum and penicillin/streptomycin as above. Cells were maintained in 5% CO_2_ and either normoxia (ambient 21% O_2_) or in hypoxia (Invivo_2_ 400 workstation, Ruskinn Technology Ltd) at 1% O2. Dimethyloxalylglycine (DMOG #D3695, Sigma-Aldrich) was added at a working concentration of 1 mM for 2 h prior to sample collection. For the HIF-inhibitor experiments, HUVECs were seeded into 6-well plates and treated with 10 μM KC7F2 (Selleckhem, S7946) or 10 μM PT2385 (Selleckhem, S8352), followed by 8 h hypoxia treatment.

### Human samples

Human vascular samples (femoral artery atherectomy samples and normal mammary arteries) were collected during a previous study (75) and two additional samples were included in the analysis of this study. Atherosclerotic samples were collected during vascular atherectomy operations and normal samples were trimmed ends collected from cardiac bypass surgery. The studies were conducted in accordance with the ethical standards laid down in the Declaration of Helsinki and approved by Local Ethical Committee of Kuopio University Hospital (53/2011). All the subjects gave informed consent.

### RNA isolation and quantitative reverse transcription PCR

Total RNA from cells was isolated using RNeasy Kit (Qiagen) followed by DNase treatment using the Turbo DNase kit (Ambion). Total RNA was used for complementary DNA (cDNA) synthesis using SuperScript IV Reverse Transcriptase (Invitrogen) primed with 1.25 μM random hexamer primers in combination with 0.5 μM tagged strand-specific primers (76). Strand specificity was monitored using control reverse transcription (RT) reactions omitting the RT primers to detect amplification of spuriously primed cDNA. To exclude genomic DNA contamination, a minus reverse transcriptase control samples were prepared. No-template controls were used in the quantitative PCR (qPCR) to ensure specific amplification products. Quantitative transcript analysis was performed on a Step One Plus qPCR machine using Power SYBR Green or TaqMan Gene Expression PCR Master Mix (Applied Biosystems). Melt curve analysis and agarose gel visualization was used to ensure specific product amplification. Custom primers and probes (Table S1) were synthesized by Integrated DNA Technologies. Values are normalized to ATP5F1 or RPLP0 mRNA, which were confirmed to have hypoxia stable expression using RNA-seq. The ΔΔ*C*_T_ method was used to determine the relative expression of RNA.

### Western blot

Proteins were isolated using NE-PER Nuclear and Cytoplasmic Extraction Reagents (Thermo Fisher Scientific) Nuclear protein extract was purified from frozen HUVEC and EAhy cells using NE-PER Nuclear and Cytoplasmic Extraction Reagents (Thermo Fisher Scientific #11865694). The lysis buffer for protein extraction was supplemented with cOmplete, EDTA-free Protease Inhibitor cocktail (Roche #11873580001). Protein concentration was quantified using BCA protein assay kit (Thermo Fisher Scientific #10741395). Following quantification, 15 μg of nuclear protein from EAhy cells and 5 μg from HUVEC cells was mixed in equal volume of 2× Laemmli Sample Buffer (Bio-Rad #1610737). The mix was heated at 95 °C for 5 min after which it was separated by electrophoresis using 4 to 15% Mini-PROTEAN TGX Precast Protein Gels (Bio-Rad #4561084 and #4561085). The separated proteins were transferred to Trans-Blot Turbo Transfer Pack, nitrocellulose, 7 × 8.5 cm (Bio-Rad #1704158). The membranes were blocked using 5% skimmed milk in Tris-*buffered* saline-Tween for 1 h at RT and incubated with primary antibody HIF-1a (1:500; Novus Biologicals; NB-100479) for overnight at 4 °C. Subsequently the membranes were incubated with mouse anti-rabbit IgG-HRP (1:1000; Santa Cruz Biotechnology; sc-2357) secondary antibody for 1 h at RT. HDAC1 (1:1000; Cell Signaling Technology; CST-5356S) was used as loading control along with Anti-mouse IgG, HRP-linked Antibody (1:1000; Cell Signaling Technology; CST-7076P2) following similar protocol as above. Signal was developed using Pierce ECL Plus Western Blotting Substrate (Thermo Fisher Scientific #32132) and visualized with Bio-Rad ChemiDoc MP imaging system. Western blot quantification was performed using ImageJ (https://github.com/imagej).

### Antisense oligonucleotide knockdown and transfections

Three GapmeR locked nucleic acid antisense oligonucleotides (ASOs; Table S1) were designed and synthesized by Exiqon as was NegA which was used as a negative control. The ASOs were diluted in serum free media and transfected in HUVECs using Lipofectamine 2000 for 4 h before being washed and exchanged for EGM and collected 48 h after transfection. An optimized final concentration of 20 nM was used for all ASOs.

### RNA FISH and microscopy

HUVECs were cultured on 8-well chamber slides (BD Falcon CultureSlides) in both hypoxic (8 h) and nonhypoxic conditions as described earlier. RNA FISH was performed by using ViewRNA Cell Assay (Affymetrix) according to the manufacturer’s instructions, deviating with addition of 1% acetic acid to the 4% paraformaldehyde in PBS to enable nuclei permeability. Specific probe sets for the *HIF1A-AS* and the nuclear lncRNA *MALAT1* (#VA1-11317) used as a positive control for nuclear staining were designed by Affymetrix. Region chr14:62196203-62198596 was used in probe design for *HIF1A-AS*. Microscopy and analysis were performed using a Zeiss LSM700/LSM800 confocal microscopes with ZEN 2012 software (https://www.zeiss.com/microscopy/en/products/software/zeiss-zen.html). Z-stacks were processed into maximum intensity projections and were analyzed using ZEN Image Analysis software (v. 3.5). Analysis was performed on a total of 498 cells from 16 (normoxia) and 23 (hypoxia) images. Automatic image segmentation was performed using Zones of Influence method to detect individual nuclei (DAPI signal) and RNA FISH-stained objects (HIF1A-AS probe fluorescence) within the nuclei. Objects were detected using Global Thresholding (Click method) using minimum intensity of 5 for nuclei and 30 for RNA FISH objects. Pick behavior settings: tolerance 1% for nuclei, 3 % for RNA FISH objects, minimum size of 1000 px for nuclei, and 3 px for RNA FISH objects. Nuclei were further filtered for minimum size of 100 µm^2^.

### CRISPR/Cas9 mediated induction and deletion of *HIF1A-AS*

The mammalian guide expression vector pSPgRNA was a gift from Charles Gersbach (Addgene plasmid # 47108; http://n2t.net/addgene:47108; RRID:Addgene_47108) (77). The catalytically dead (d)Cas9 fused with VP64-p65-Rta (VPR) activator (SP-dCas9-VPR) was a gift from George Church (Addgene plasmid # 63798; http://n2t.net/addgene:63798; RRID:Addgene_63798) (39). Oligonucleotides for gRNA sequence targeting *HIF1A-AS* promoter were designed using CRISPR design tool (crispr.mit.edu) and ordered from Integrated DNA Technologies. Overhangs CACCG and AAAC were included to the 5′ ends of forward and reverse oligonucleotides, respectively. Oligonucleotides were phosphorylated using T4 PNK (New England Biolabs) and annealed oligos were ligated to pSPgRNA digested with BbsI restriction enzyme (Thermo Fisher Scientific). Plasmids were purified for transfections using EndoFree Plasmid Maxi Kit (Qiagen). HIF1A-AS gRNA-containing pSPgRNA and SP-dCas9-VPR plasmids were cotransfected to HEK293T cells using Lipofectamine 2000 according to manufacturer’s instructions.

For CRISPR-Cas9 ablation of the *HIF1A-AS* in EAhy.926 cells, gRNAs were designed to target the flanks of the 460 bp promoter (as determined by GRO-seq, H3K4me3 and ENCODE TFBS ChIP data). The crRNA:tracrRNA duplex was formed by denaturing 1 μM of each at 95 °C for 5 min and left to hybridize at room temperature. Equimolar quantities of Alt-R S.p. HiFi Cas9 Nuclease (Integrated DNA Technologies) and gRNA were then complexed at room temperature for 15 min. Ribonucleoprotein complexes were reverse transfected into 8 × 10^4^ EAhy.926 cells on 96 well plates using Lipofectamine CRISPRMAX (Invitrogen) according to the manufacturer’s instructions. Three independent clonal cell lines of EAhy.926 ΔHIF1A-AS were established through single cell isolation where colonies were confirmed to have the *HIF1A-AS* deletion using PCR with primers targeting the *HIF1A-AS* promoter flanks and visualization with gel electrophoresis combined with sanger sequencing.

### RNA-seq sample preparation and data analysis

RNA-Seq and GRO-Seq samples for HUVECs treated with hypoxia or adenoviral overexpression of HIF1/2A were collected as part of earlier studies (3, 37, 78). GRO-Seq samples from normoxia or hypoxia-treated (8 h) HAECs, HASMCs, and CD14+ macrophages were prepared as described (79). GRO-Seq samples from HEK293T cells cotransfected with control plasmid (pSPgRNA) or HIF1A-AS targeting *gRNA-*pSPgRNA and SP-dCas9-VPR were prepared as described 24 h posttransfection (79). Public GRO-Seq datasets from 12 additional cell types used in correlating *HIF1A-AS* and *HIF1A* expression levels were processed as described earlier (80). RNA from clonal cell lines of EAhy.926 ΔHIF1A-AS was isolated using RNeasy minikit (Qiagen) and DNase treated with the RNase-free DNase set (Qiagen). RNA library preparation was carried out using a NEBNext Ultra II directional RNA library prep kit for Illumina (New England BioLabs) according to the manufacturer’s instructions. The resultant library was quantified using a Qubit double-stranded-DNA HS assay kit (Thermo Fisher Scientific), and quality checked with a Bioanalyzer (Agilent Technologies). Individual libraries were pooled in equimolar amounts (4 nM for each) and sequenced with the NextSeq 550 sequencer (Illumina) using 75 single-end cycles.

RNA-seq and GRO-seq reads were preprocessed by trimming and filtering using Trim Galore (v.0.4.4) with a Phred quality score cutoff 30. Processed RNA-seq reads were aligned to GRCh37 using STAR version 2.5.4 b (81) with options --outFilterMismatchNoverLmax 0.04 and --outFilterMultimapNmax 10. GRO-seq reads were assigned using bowtie2/2.3.4.1 using the best match with a two-mismatch tolerance. Detection of novel lncRNAs from GRO-seq data was performed by using Homer v4.9 (82) command “findPeaks” with -GRO seq option. Novel lncRNAs were concatenated with GENCODE v19 GTF and filtered to remove duplicated transcripts. RNA-seq Reads were assigned using featureCounts (Rsubread 1.32.4) (83). Expression data were filtered using edgeR::filterByExpr, to retain genes with >10 counts per sample for at least 70% of a given grouping. Library sizes were subsequently normalized using trimmed mean of M values and the generalized linear model likelihood ratio test was used to estimate differential expression using edgeR 3.24.3 (84). Transcripts with a log_2_ FC of ≥ 0.7 or ≤ −0.7 and false discovery rate < 0.01 were considered differentially expressed. Genome browser shots were created using UCSC genome browser using representative bigWigs created using the WiggleTools (1.2.10) mean function from trimmed mean of M values normalized bigWig files created using deeptools2 (3.5.1) (85). Plots were produced and visualized in R using ggplot2 (86). Multidimensional *scaling* for sample quality controls was performed using classical multidimensional *scaling function.*

Expressed antisense transcripts overlapping protein coding genomic regions were identified using BEDtools (87) intersect with -S option with at least 10% overlap for the antisense. To quantify *HIF1A-AS* expression, instead of focusing on the 2051 bp *HIF1A-AS2* (NR_045406) coordinates, *HIF1A-AS* expression was detected using the coordinates of the full-length transcript detected in this study (chr14:62,162,559–62,217,816). To quantify intronic and exonic signals from the *HIF1A* gene, analyzeRepeats.pl program from Homer software (http://homer.ucsd.edu/homer/) was utilized with-count exons and with-count introns options. Due to high amount of vector-derived signal in AdHIF1a RNA-Seq samples, the 3′UTR sequence from exon 15 (which are absent in the AdHIF cDNA constructs) were used to quantify endogenous expression from *HIF1A* in AdHIF-treated samples. For quantifying intronic signal from AdHIF samples, location of intron 1 (chr14:62,163,120–621,868,05) was manually selected to avoid signal originating from exonic reads.

### ChIP-seq (H3K4me3)

ChIP-seq libraries were prepared as previously described (79). Briefly, 1% formaldehyde fixed cells were collected, sonicated, and H3K4me3 complexes were precipitated using rabbit polyclonal antisera to H3K4me3 (Abcam, #ab8580) with rabbit IgG used as a negative control. DNA was purified, blunted, and used for library preparation with ligation to NEXTflex DNA barcode adapters and indexes (Bioo Scientific Corporation) were incorporated and amplified using PCR. Library fragments were size selected on 10% polyacrylamide gel and sequenced on a NextSeq 500 (Illumina) using single-end 75 cycles in high output mode.

### HT-ChIPmentation (H3K27ac)

For high throughput ChIPmentation, clonal cell lines of EAhy.926 ΔHIF1A-AS were seeded into 15-cm plates and cultured in hypoxic (8 h) and nonhypoxic conditions. The cells were washed once with PBS and subsequently fixed with 1% formaldehyde for 10 min at room temperature. The fixed cells were washed twice with ice-cold 1 × PBS containing 1 × protease inhibitors. The cells were scraped and suspended in 6 ml of ice-cold Farnham Lysis Buffer (5 mM Pipes pH 8, 85 mM KCl, 0.5% IGEPAL, 1 × proteinase inhibitor cocktail). The cell suspension was then centrifuged at 2000 rpm for 5 min at 4 ˚C. The cell pellet was resuspended in ice-cold 1 ml Farnham Lysis Buffer, mixed gently, and incubated on ice for 10 min. The crude nuclear prep was collected by centrifugation at 2000 rpm at 4 ˚C for 5 min. The pellet was resuspended in 600 μl ice-cold radioimmunoprecipitation assay buffer (1xPBS, 1% IGEPAL, 0.5% sodium deoxycholate, 0.1% SDS, 1 × proteinase inhibitor cocktail) for sonication. Chromatin was fragmented with Bioruptor Plus sonicator (Diagenode) for 35 cycles at high setting. Each cycle was 30 s ON, 30 s OFF, and after each 10 cycles the sonicator was allowed to cool for 5 min. To neutralize the SDS, Triton-X-100 was added to a final concentration of 1% along with 50× protease inhibitors (final 1×). After centrifugation at 16,000 rpm for 20 min, the supernatant containing the sheared chromatin was collected, and a 20 μl aliquot was taken for the preparation of the input control. The remaining sample was divided into 200 μl aliquots for immunoprecipitation.

For the ChIP and library preparation, the ChIPmentation protocol described by Gustafsson *et* *al*. (88) was followed with minor modifications. For the ChIP step, either 5 μg of anti-histone H3 (tri methyl K36) antibody (#ab9050, Abcam) or 5 μg of Anti-Histone H3 (acetyl K27) antibody (#ab4729, Abcam) was added to 50 μl of Protein G-coupled Dynabeads (Thermo Fisher Scientific) in 1 × PBS with 0.5% bovine serum albumin and rotated at 4 °C for 4 h. The antibody-coated Dynabeads were washed three times with PBS containing 0.5% bovine serum albumin, then mixed with 200 μl of the sonicated chromatin samples and rotated overnight at 4 °C. The immunoprecipitated chromatin was subjected to five washes with LiCl immunoprecipitation wash buffer (100 mM Tris pH 7.5, 500 mM LiCl, 1% IGEPAL, 1% sodium deoxycholate, and 1 × proteinase inhibitor cocktail), followed by two washes with Tris-EDTA buffer (10 mM Tris–Cl and 1 mM EDTA), and two washes with Tris–HCl pH 8.

Immunoprecipitated chromatin bound to beads was resuspended in 30 μl of 1 × Tagmentation buffer with 1 μl of Tagmentase (loaded, Diagenode) for tagmentation at 37 °C for 10 min The reaction was terminated by adding radioimmunoprecipitation assay buffer and the beads were washed twice with 10 mM Tris–HCl pH 8.0. Bead-bound tagmented chromatin was diluted in 21 μl of water and 25 μl of 2 × NEBNext High-Fidelity 2X PCR Master Mix (New England Biolabs) and indexed amplification primers (24 UDI for Tagmented libraries, Diagenode) were added to the final concentration of 0.125uM. For adapter extension and reverse cross-linking, the libraries were incubated as follows: 72  °C 5 min (adapter extension); 95  °C 5 min (reverse cross-linking); followed by 12 cycles of 98  °C 10 s, 63  °C 30s and 72  °C 3 min. After PCR amplification, libraries were purified using SPRIselect beads (Beckman Coulter). To prepare input controls for sequencing, 20 μl of sonicated chromatin was used. Tagmentation and amplification was done as described above. The library yield was quantified with Qubit DNA High Sensitivity assay (Invitrogen) and quality control was performed on the Agilent Bioanalyzer with High Sensitivity DNA kit (Agilent). ChIP-seq libraries were sequenced on the NextSeq 500 (Illumina) with single-end 75 bp-reads.

### Metabolic labeling

For metabolic labeling of nascent RNA, Click-iT Nascent RNA Capture Kit (Invitrogen, #C10365) was used according to the manufacturer’s instructions. Briefly, cells were pulse labeled by adding 5-EU to a final concentration of 500 μM between 15 to 60 min. RNA was isolated using TRIzol reagent (Invitrogen) with 20 μg glycogen (#R0551, Thermo Fisher Scientific) with the nascent biotinylated RNA captured on streptavidin T1 beads. On-bead reverse transcription was performed using the previously described methodology. Single-strand cDNA was eluted with the addition of 2.5 U RNaseH and incubated at 37 °C for 15 min and inactivated at 80 °C for 10 min. The eluent was removed from the beads, diluted, and used for downstream quantitation or library preparation.

### Figures and statistical analysis

Statistical analysis was performed using GraphPad Prism version 5.03 (https://www.graphpad.com/) or R version 3.6 (https://www.r-project.org/). Plots were prepared using GraphPad Prism, ggplot2 and ComplexHeatmap (89). UCSC Genome browser was used to visualize genomic regions. Figure layout, scaling, and font adjustments were performed using Adobe Illustrator CS5 version 15.0.0. Statistical tests are performed as stipulated in the text; tests for relative expression ratios obtained from qPCR are performed on delta *C*_T_ values.

## Data availability

The GRO-Seq and RNA-Seq samples for HUVECs treated with hypoxia or adenoviral overexpression of HIF1/2a can be found under accession numbers GSE94872 and GSE98060. GRO-Seq samples from normoxia or hypoxia-treated (8 h) HAECs, HASMCs, and CD14+ macrophages can be found under accession numbers GEO1552744.

Public GRO-Seq datasets from additional cell types were downloaded from GEO under accession numbers GSM1014633, GSM1240748, GSM1014636, GSM1014635, GSM1240742, GSM1014634, GSM1240741, GSM1240749, GSM1240738, GSM1240746, GSM1240747, GSM1014631, GSM1240739, GSM1014632, GSM980644, GSM980645, GSM1480326, GSM340902, GSM1648604, GSE145568, GSE120886, GSM1648614, and GSM1524923 were used in correlating HIF1a-AS and HIF1a expression levels. GRO-Seq datasets were processed as described earlier. The data generated as part of this study is available in GEO under accession number GSE221387.

## Supporting information

This article contains supporting information.

## Conflict of interest

The authors declare that they have no conflicts of interest with the contents of this article.
