# Supplementary material for: Hypoxic regulation of hypoxia inducible factor 1 alpha via antisense transcription
Source: J Biol Chem. 2023 Sep 23;299(11):105291. doi: 10.1016/j.jbc.2023.105291 (PMC10630634; doi:10.1016/j.jbc.2023.105291)
Supplement: Supplementary information [file mmc1.pdf]

Supplementary Information

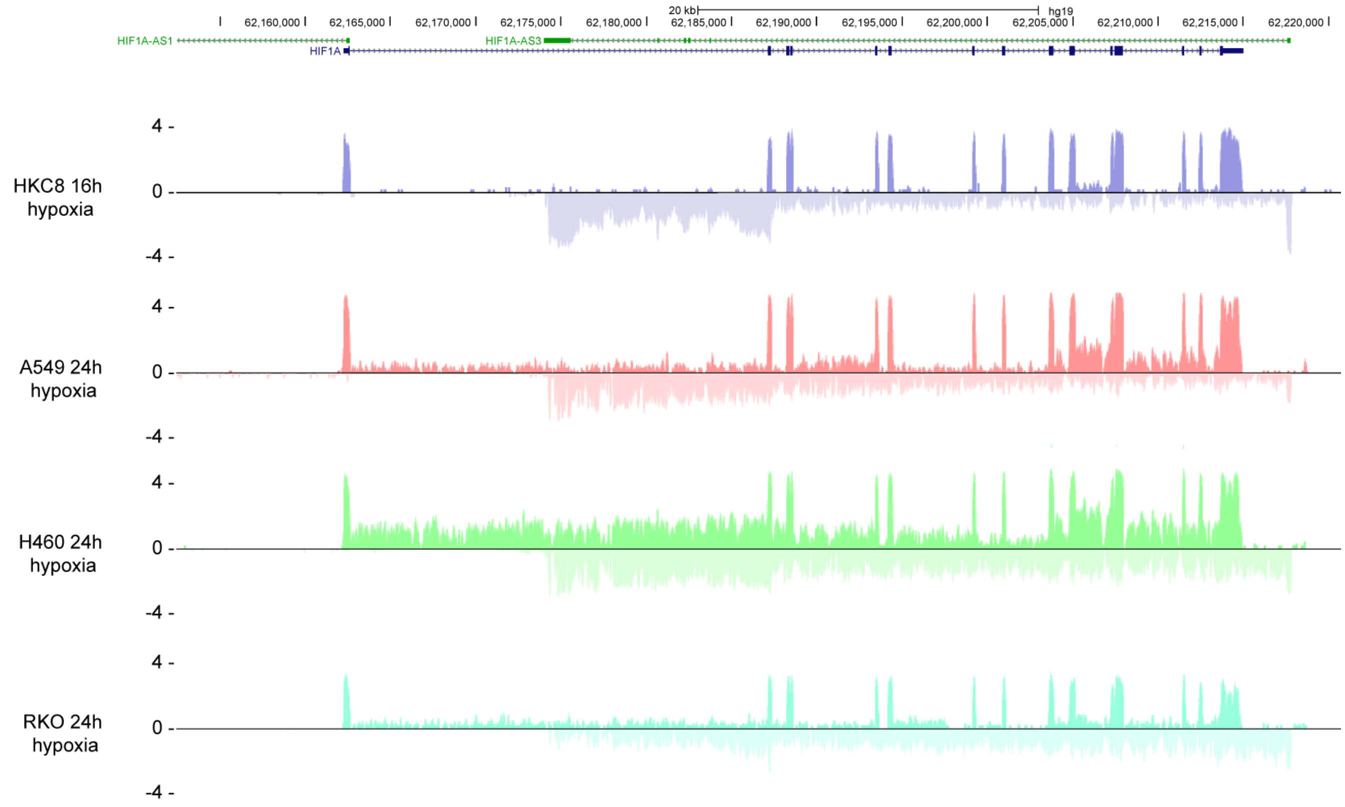

Figure S1: Genome browser tracks showing RNAseq alignments from hypoxic HKC8, A549, H460 and RKO cells (from GSE145568 and GSE120886). Y-axis is log transformed (ln1+x).

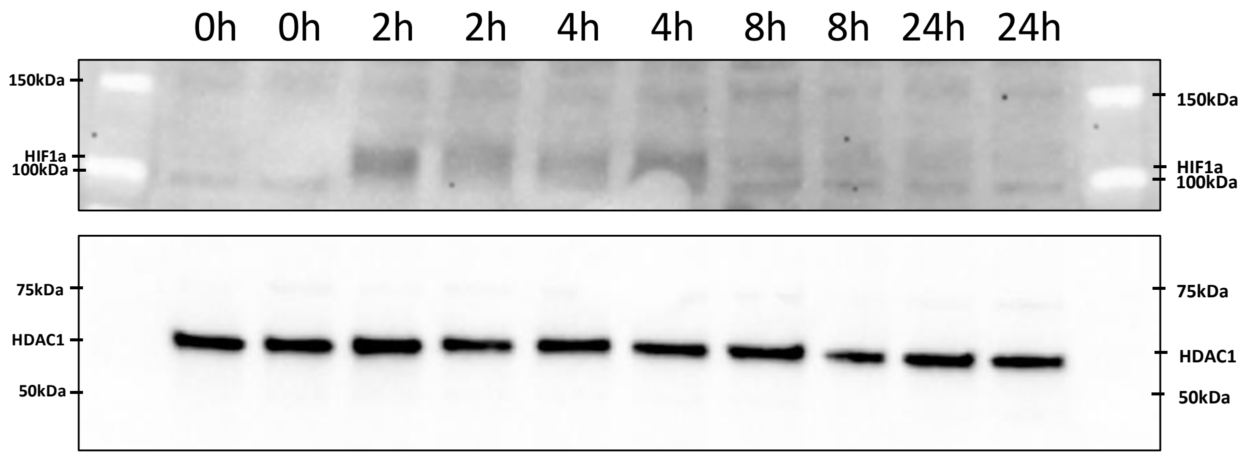

Figure S2: Western blot for HIF1A protein isolated from HUVEC nuclear extracts at multiple hypoxia time points. HDAC1 used as a loading control.

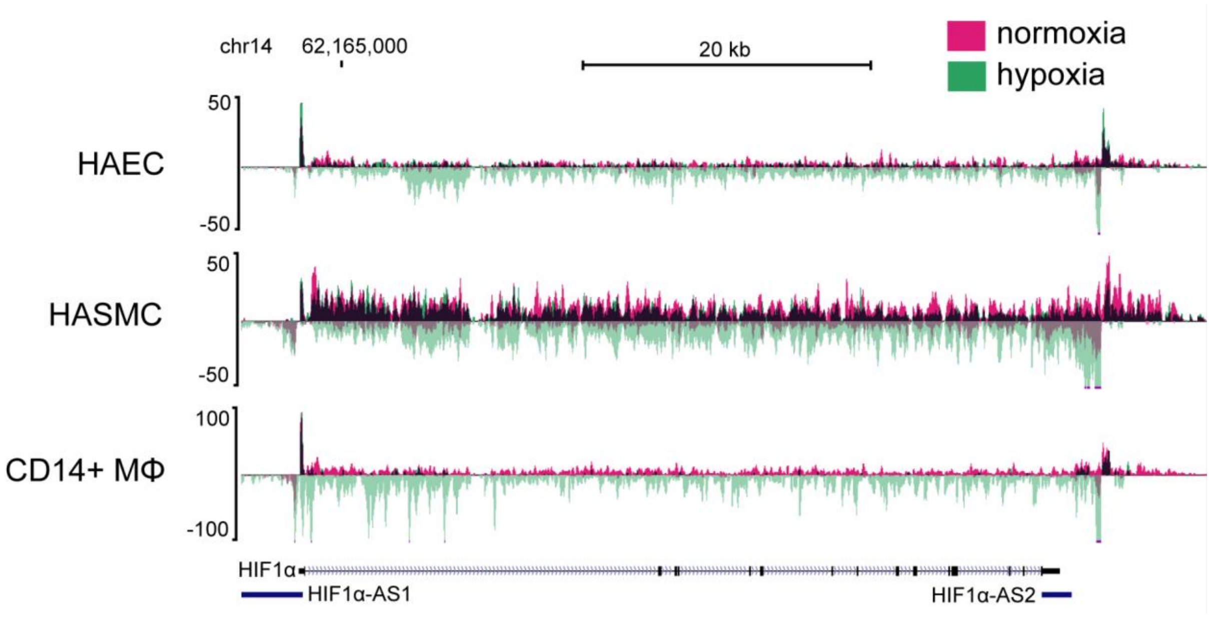

Figure S3: UCSC browser image of the *HIF1A* locus in normoxia and hypoxia in HAECs, HASMCs and CD14+ macrophages (MΦ). Normalized reads for GRO-Seq samples are presented.

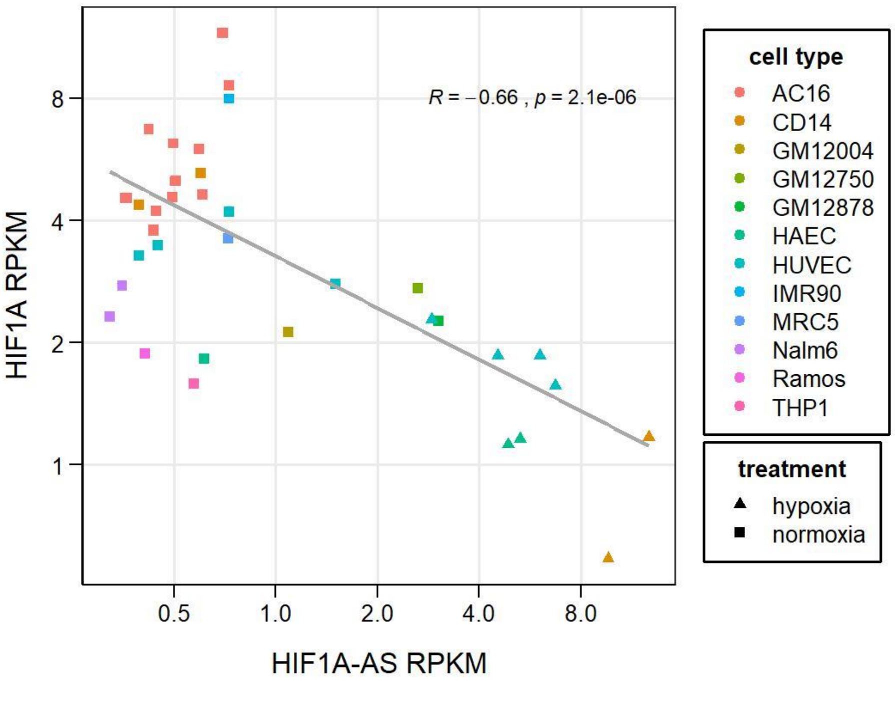

Figure S4: Pearson correlation of *HIF1A* and *HIF1A-AS* nascent transcript expression in 12 cell types using GRO-Seq.

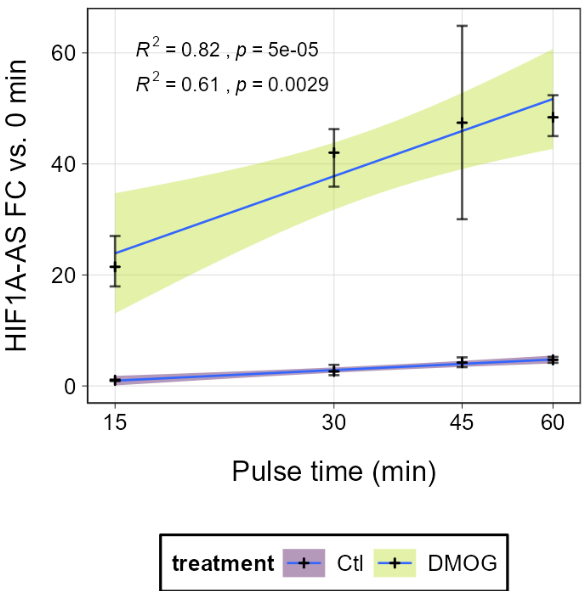

Figure S5: Relative expression of *HIF1A-AS* labelled with 5-Ethynyl Uridine for 15, 30, 45 or 60 minutes in untreated and dimethyloxallyl glycine (DMOG) treated HUVECs, error line represents 95% confidence intervals.

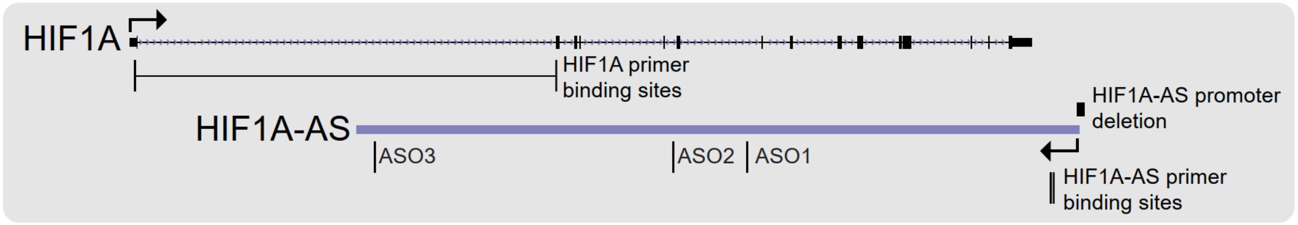

Figure S6. Schematic representing the annotation of *HIF1A* and *HIF1A-AS*. The locations of the 3 ASO targeting the *HIF1A-AS* transcript, site of promoter deletion and primer sites used to quantify *HIF1A-AS* and *HIF1A* expression via qPCR are shown.

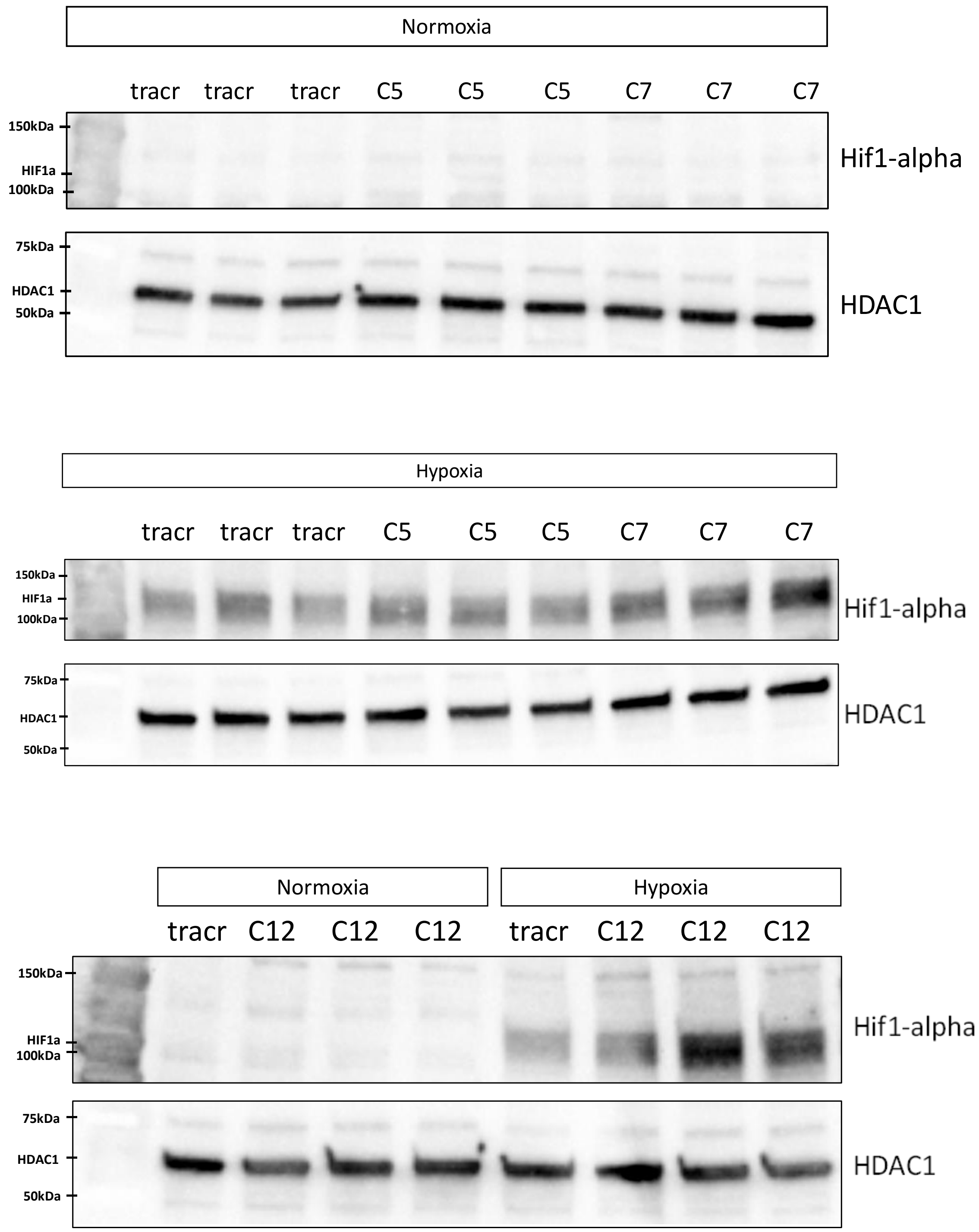

Figure S7: Western blot for HIF1a protein isolated from nuclear extracts of hypoxic and normoxic treated wildtype and CRISPR modified (ΔHIF1A-AS, clones C05, C07 and C12 compared to tracer-only control cells) EAhy cells. HDAC1 used as a loading control.

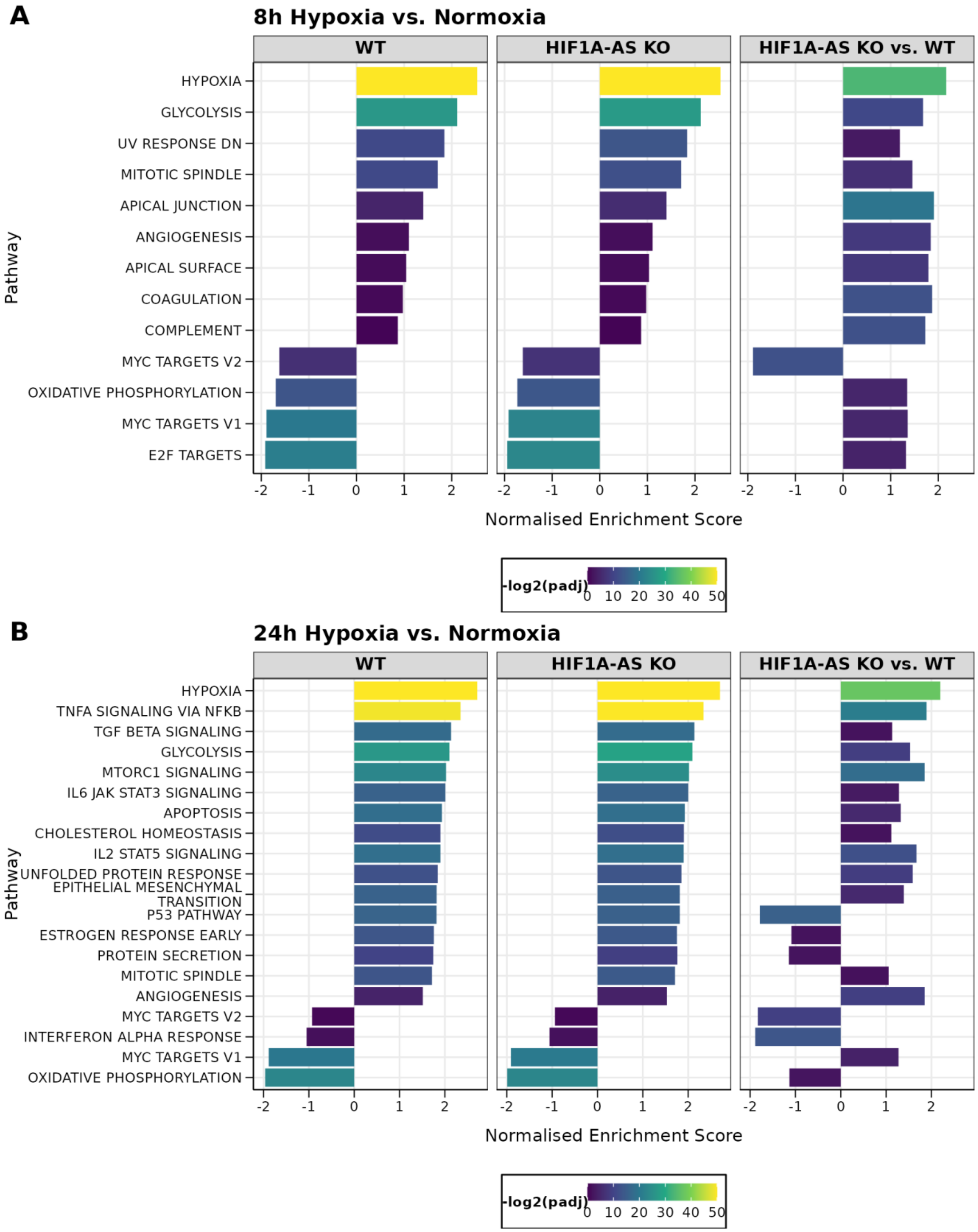

Figure S8: Gene set enrichment analysis (GSEA) for genes differentially expressed in **(A)** 8h of hypoxia and **(B)** 24h of hypoxia, against MSigDB hallmark gene sets. Groups shown include wildtype and HIF1A-AS knockout EA.hy cell lines, and differential expression of HIF1A-AS knockout cells relative to wildtype cells in the respective hypoxia time-point.

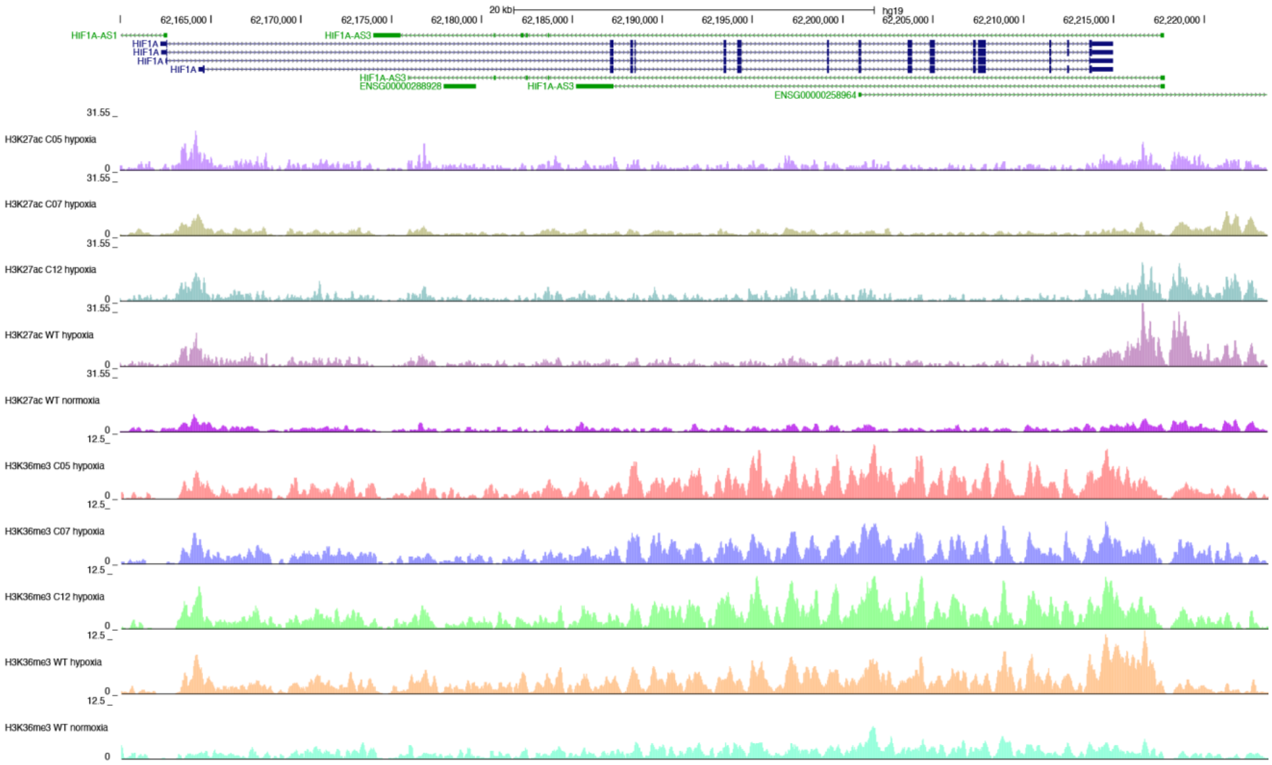

Figure S9: UCSC browser track showing normalised H3K36me3 and H3K27ac ChIPseq reads from the *HIF1A* locus from wildtype and *HIF1A-AS* knockout cell lines (C05, C07, C12) in hypoxia. Levels of H3K36me3 and H3K27ac in wildtype cells are shown for reference.

Target	*ID or sequence*
Control scrambled ASO	AACACGTCTATACGC, LNA longRNA GapmeR *in vivo* ready, Exiqon 300612-00
HIF-1-AS ASO1	GTGTAGATGCTCTTTG, LNA longRNA GapmeR *in vivo* ready, Exiqon
HIF-1-AS ASO2	GAAGTAGGTCATAGG, LNA longRNA GapmeR *in vivo* ready, Exiqon
HIF-1-AS ASO3	GTTAGCATGAGAAAC, LNA longRNA GapmeR *in vivo* ready, Exiqon
HIF1A-ASgRNA	Fwd: CACCGTACACGAAAGTCGCCTTGCG,
	Rev: AAACCGCAAGGCGACTTTCGTGTAC
HIF1A-AS	Fwd: TGACGGTGCATAAAATTGGGAAG,
	Rev: TGAAAGCATTGAGAAGGGAAAGC
HIF1A Exon1-Exon2	Fwd: TTTCCTTCTCTTCTCCGCGT
	Rev: CGAGACTTTTCTTTTCGACGTTC
	Probe: CCGCCCGCCGTGAAGACATCGC
RPLP0	Fwd: GGAGACGGATTACACCTTCCC
	Rev: CAGCCACAAAGGCAGATGG
ATP5F1	Fw:GCCCTGACAGATTCTCCTATCG,
	Rev:GAAAGGTCCTTGTTGCCTGC
HIF1A Exon 2 tagged SPRT	GGCAGTATCGTGAATTCGATGCCGAGACTTTTCTTTTCGACGTTC
Tag Primer Rev	aataaatcataaGGCAGTATCGTGAATTCGATGC*
HIF1A Intron1-Exon2 Tagged Fwd	aataaatcataaTGGCATCTTCTAATCCTTCTGTG*
HIF1A-AS crRNA 5'	CUUCUGGGACUUGUCAAAGUGUUUUAGAGCUAUGCU
HIF1A-AS crRNA 3'	UUCAAUAGUACACGGAGAUCGUUUUAGAGCUAUGCU
HIF1aAS TSS CRISPR screen	Fwd: TTTCAAGTGCCATTTAGTGGGTTC
	Rev: CAGCTTGTTTCTGTACGCCTATG
ChIP-seq adapters	Bioo Scientific NEXTflex 6 DNA Barcoded Illumina Adapters #514101
*oNTI200-Index***	CAAGCAGAAGACGGCATACGAGATXXXXXXGTGACTGGAGTTCAGACGTGTGCTCTTCCGATCT
oNTI201-Index	AATGATACGGCGACCACCGACAGGTTCAGAGTTCTACAGTCCGACG
oNTI223-Index	/5Phos/GATCGTCGGACTGTAGAACTCTGAAC/iSp18/TCAGACGTGTGCTCTTCCGATCTTTTTTT TTTTTTTTTTTTTVN
Illumina P5	AATGATACGGCGACCACCGA
Illumina P7	CAAGCAGAAGACGGCATACGA
	*lowercase nucleotides denotes noncomplementary 5' overhangs<br>**xxxxxx 6nt barcode

Table S1: Oligonucleotides used in this study
